# Improving detection and initial management of gestational diabetes through the primary level of care in Morocco: protocol for a cluster randomized controlled trial

**DOI:** 10.1186/s12978-017-0336-z

**Published:** 2017-06-19

**Authors:** Bettina Utz, Bouchra Assarag, Amina Essolbi, Amina Barkat, Nawal El Ansari, Bouchra Fakhir, Alexandre Delamou, Vincent De Brouwere

**Affiliations:** 1 Institute of Tropical Medicine, Antwerp, Belgium; 2 National School of Public Health, Rabat, Morocco; 3 Faculty of Medicine and Pharmacy, Mohammed V University, Rabat, Morocco; 4 Faculty of Medicine, Cadi Ayyad University, Marrakesh, Morocco; 5 Maferinyah Training and Research Centre, Conakry, Guinea

**Keywords:** Gestational diabetes, Screening, Management, Primary health care, Maternal health, Morocco, Implementation, Protocol

## Abstract

**Background:**

Morocco is facing a growing prevalence of diabetes and according to latest figures of the World Health Organization, already 12.4% of the population are affected. A similar prevalence has been reported for gestational diabetes (GDM) and although it is not yet high on the national agenda, immediate and long-term complications threaten the health of mothers and future generations. A situational analysis on GDM conducted in 2015 revealed difficulties in access to screening and delays in receiving appropriate care. This implementation study has as objective to evaluate a decentralized GDM detection and management approach through the primary level of care and assess its potential for scaling up.

**Methods:**

We will conduct a hybrid effectiveness-implementation research using a cluster randomized controlled trial design in two districts of Morocco. Using the health center as unit of randomization we randomly selected 20 health centers with 10 serving as intervention and 10 as control facilities. In the intervention arm, providers will screen pregnant women attending antenatal care for GDM by capillary glucose testing during antenatal care. Women tested positive will receive nutritional counselling and will be followed up through the health center. In the control facilities, screening and initial management of GDM will follow standard practice. Primary outcome will be birthweight with weight gain during pregnancy, average glucose levels and pregnancy outcomes including mode of delivery, presence or absence of obstetric or newborn complications and the prevalence of GDM at health center level as secondary outcomes. Furthermore we will assess the quality of life /care experienced by the women in both arms. Qualitative methods will be applied to evaluate the feasibility of the intervention at primary level and its adoption by the health care providers.

**Discussion:**

In Morocco, gestational diabetes screening and its initial management is fragmented and coupled with difficulties in access and treatment delays. Implementation of a strategy that enables detection, management and follow-up of affected women at primary health care level is expected to positively impact on access to care and medical outcomes.

**Trial registration:**

The trial has been registered on clininicaltrials.gov; identifier NCT02979756; retrospectively registered 22 November 2016.

## Plain English summary

A situational analysis on gestational diabetes in Morocco revealed that pregnant women are experiencing difficulties in accessing GDM screening coupled with delays in receiving appropriate care for those affected. To evaluate a decentralized GDM detection and management approach through the primary health care level in Morocco, we are conducting a hybrid effectiveness-implementation research using a cluster randomized controlled trial design in two districts. In each district, 10 health centers were randomly selected with half of them serving as intervention and half as control facilities. In the intervention arm, women attending antenatal care will be screened for GDM and women positively tested will receive nutritional counselling and follow up through their health centers. In the control facilities, screening and initial management of GDM will apply standard practice. Birthweight of newborns of women with GDM will be compared between both groups and the prevalence of GDM at health center level assessed. We will further explore the quality of life and the care experienced by the women in both arms through a structured survey. In focus group discussions with providers we will explore and evaluate the feasibility of the intervention at primary level and its adoption by the health care professionals.

The findings of this study will be used to inform national stakeholders and are expected to contribute to an adaptation of the current strategy of detection and management of GDM in Morocco.

## Background

In Morocco, a country with a total population of 33.8 million, an estimated 1.67 million people suffer from diabetes [1]. With a current prevalence of diabetes in Morocco of 7.7%, this number is expected to double in the next 20 years. [1] Women of childbearing age are not exempted from this development and alone in the MENA region, 3.7 million babies are born to mothers affected by gestational or pre-existing diabetes [1]. The prevalence of gestational diabetes (GDM) in Morocco is estimated to range between 8.2 and 10% [2, 3]. These figures originate from prevalence studies conducted at two university hospitals, but prevalence rates at lower levels of care are yet unknown. While Morocco has made considerable progress in lowering maternal mortality in the past decades [4], there is now an increased focus on maternal morbidity, including the effects of non-communicable diseases such as gestational diabetes on maternal and neonatal health [5].

Recommendations of high risk pregnancy management including the detection of GDM are available in Morocco and a fasting blood glucose test is routinely recommended to screen for diabetes during antenatal care (ANC). However, a situation analysis indicated that GDM screening is facing various obstacles particularly at the primary level of care. [6] These include gaps in provider knowledge, delays related to blood testing or to specialist referral. Oral glucose tolerance tests are not routinely prescribed unless on specific request. These delays in the detection and treatment of GDM impinge a timely management of affected women who as a result may end up with complications that could have been prevented. The detection and initial management of GDM during ANC through the first level of care could be an appropriate approach to establish timely interventions. However, it is uncertain if such a strategy would be feasible in the context of the Moroccan public health sector. To close this knowledge gap, the study presented in this protocol aims at implementing and evaluating GDM screening and follow up through first line health care services in Morocco.

## Methods/design

### Design

We opted for a hybrid effectiveness-implementation research using a cluster randomized controlled trial design. This design allows to evaluate both clinical effectiveness of the proposed strategy and its implementation at the first level of care guided by its potential for national scaling up [7].

### Study setting

The proposed study will take place in the region of Marrakech-Safi, a region with poor maternal health indicators and one of the priority regions of the Moroccan Ministry of Health. The two study districts Marrakech and Al Haouz, where we already conducted a situational analysis on GDM in 2015 [6], have been selected for the strategy implementation. Marrakech is essentially urban whereas Al Haouz is a rural mountainous district with difficult geographical access to services. Together, the districts have a total population of 1.9 million with 92 public health centres and 53 dispensaries providing primary health care services [8].

### Unit of randomization

The unit of randomization will be the health center (cluster). Cluster randomization rather than individual randomization will be applied to limit contamination between the different arms. In each of the two districts we will randomly select 10 health centers with at least 30 or more antenatal care consultations a month. Of these 10 health centers in each district, 5 are being randomized into the intervention group and 5 health centers serve as controls. As simple random selection does not account for contiguity, adjustments for “inter-cluster” effects will be done in the analysis stage.

### Population and sample size

All pregnant women attending ANC will be eligible to participate in the study. Pregnant women with a known diabetes type 1 or 2 will be excluded from participation. In each health center, at least eight women diagnosed with gestational diabetes will be identified and followed up during their pregnancies and up to eight weeks post-partum. The sample size of minimum eight women per cluster was calculated based on the assumption that neonatal birth weight will differ in both groups with lower birthweights in the intervention arm due to earlier screening and treatment initiation at health center level. Based on comparisons of birthweight in treated versus untreated mothers [9, 10], for the assumption of a weight difference of 300 g between intervention and control group, a power of 80%, an alpha of 0.05 and an intra-class correlation coefficient of 0.1, we require 75 women with GDM in each arm.

A subsample of four women per district and arm will be selected for in-depth interviews based on the their educational level (≤primary / ≥secondary); and distance from the health center (<30 min / >1 h). Furthermore, the health care providers from both intervention and control facilities who are involved in GDM detection and management will be requested to participate in a focus group discussion (2 FGDs per district).

### Intervention

In the intervention facilities, all women attending ANC will be offered a fasting glucose test at their first ANC visit and a 75 g oral glucose tolerance test (OGTT) if they are between 24 and 28 weeks pregnant. Tests will be done directly at the health center by capillary glucose testing using a standard plasma-calibrated glucometer. This corresponds to the latest consensus recommendations of FIGO for settings where no laboratories are available [11].

**Fig. 1:**
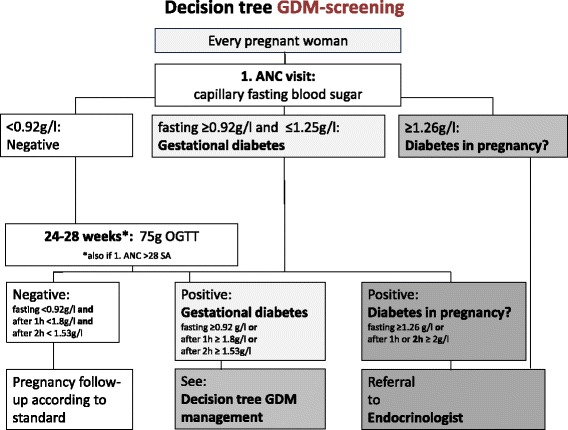
GDM screening algorithm

**Fig. 2:**
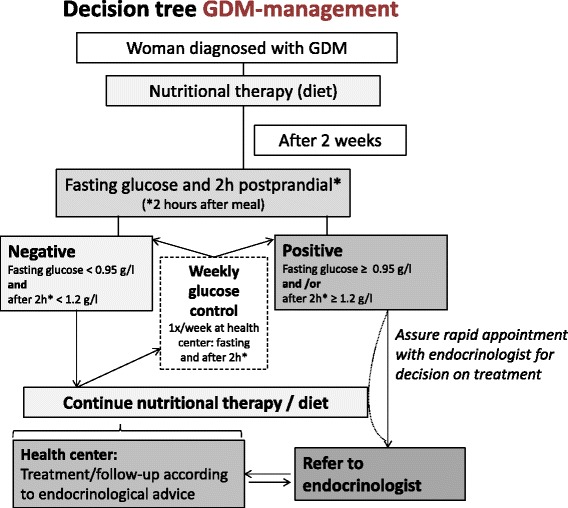
GDM management algorithm

Women who are being diagnosed with gestational diabetes in the intervention facilities will receive nutritional counselling to start first a nutritional therapy combined with regular exercise for the duration of two weeks [12]. Standardization of nutritional counselling will be supported by an initial training of intervention site providers on nutritional education and accompanied by a nutritional guideline and a patient brochure for GDM in French and Arabic developed by the Moroccan Ministry of Health. In case dietary measures will not be sufficient to control glucose values within two weeks, women will be referred to an endocrinologist for further management. Follow-up will be assured through the health centers in close collaboration with the public endocrinologists. To standardize the procedure of screening, initial management and follow-up in the intervention facilities, study algorithms that are based on both nationally and internationally recommended glycaemia thresholds for GDM testing and monitoring have been developed in consultation with the national research group on GDM and regional endocrinologists at referral level (for algorithms see Figs. 1 and 2). The health care facilities that act as controls will screen for gestational diabetes and manage affected women according to their usual practice in accordance with existing national guidelines [13].

### Outcome variables

#### Health effects

Primary outcome is birthweight to assess whether or not there is a statistically significant difference in birth weight between the two groups after adjusting for gestational age. Secondary outcomes include weight gain during pregnancy, average glucose levels and pregnancy outcomes including mode of delivery, presence or absence of obstetric (pre/eclampsia, prolonged labor, shoulder dystocia) or newborn (hypoglycemia, respiratory distress, cardiomyopathy) complications.

#### Quality of life/care

Affected women’s quality of life as well as experienced quality of care will be measured using a structured survey of all women who gave their consent to participate in this study. For this purpose, we adapted the “diabetes quality of life questionnaire” developed by Burroughs and colleagues [14].

#### Adoption of the intervention at health center level

Perceptions of providers in the intervention facilities regarding the new model of screening and initial management, the feasibility of GDM screening and follow-up at primary level, as well as facilitating factors and challenges for daily practice will be assessed through focus group discussions with providers.

#### Prevalence of GDM

Prevalence of gestational diabetes will be measured at the primary health care level using as numerator the number of women tested positive for GDM and as denominator the number of pregnant women tested.

### Data collection

Data will be collected monthly at health center level. Individual data of patients diagnosed with GDM who consented to participate in the study will be collected by data collectors based at the health facility on a daily basis in collaboration with external research assistants who will visit the facilities monthly (Table 1). Four research assistants will conduct in each facility observations of five ANC visits (first visit or between 24 and 28 weeks gestational age) to assess the information provided and the time spent on GDM. Furthermore, each woman who consented to participate in the study will be contacted post-partum by the local research assistants to conduct a structured survey on perceived quality of life/ care during her pregnancy and her expenses in relation to GDM.

**Table 1:** Quantitative data and tools

Quantitative data collection	Tools
Health center performance	
No. of ANC consultations (total and new cases) No. of women tested for gestational diabetes (GDM) (at health center level/ at the external laboratory)	Structured monthly data collection form
No. of women tested with fasting blood sugar/ oral glucose tolerance test (OGTT) No. of women diagnosed with GDM No. of women with GDM referred out to see a specialist No. of women with GDM followed up for GDM at their health facility No. of women attending postnatal care (PNC) No. of women with a GDM attending PNC	
No. of women with a history of GDM in their last pregnancy re-tested during PNC Duration ANC consultation Time dedicated to GDM (information and testing) Information provided in relation to GDM	ANC observation checklist
Individual patient information	
Socio-demographic information; Obstetric history (medical/ past obstetric problems; gravida, para, LMP, height, weight); GDM specific information: testing (date/test/site), treatment (nutritional/medical), referral and follow-up visits (interval between visits; weight, blood sugar, treatment) Information about delivery (date/ place/ mode/ newborn weight/ APGAR/ newborn or maternal complications)	Structured individual patient data collection form
Information about diabetes re-testing post-partum (date/test used) Quality of life/care Expenses in relation to GDM	Patient survey

In each district two focus group discussions with up to two providers will be conducted separately for intervention and for control facilities to explore more in-depth their perceptions and experiences with GDM detection and management. Furthermore, we will conduct in-depth interviews with a subsample of 16 women participating in this study (see sample size) to get more insight how they experienced their pregnancy and the care received. In-depth interviews with key informants such as specialists at referral level as well as national and regional maternal and newborn health programme managers on their views of the intervention, its integration into the existing system and potential for scaling up will complement the picture.

### Data analysis

Quantitative data will be double entered into a pre-formatted excel file by the research assistants and converted into STATA Version 13 for further statistical analysis. The analysis will be applied on all recruited women following the intention-to-treat principle. Comparison of categorical variables will be done by using the chi-square/ Fisher’s exact test while continuous variables will be analyzed by using the Wilcoxon rank-sum test. Regression analysis will be applied to assess the relationship between the primary outcome birthweight and maternal determinants.

Qualitative data collection includes interviews with key informants, a sample of pregnant mothers and focus group discussions with providers. The recordings will be translated (those conducted in Arabic or Berber) and transcribed verbatim, followed by a thematic content analysis. Two independent researchers will code the data using NVIVO Version 11. Codes will then be grouped into categories and findings charted into a framework matrix.

### Study management

A study advisory committee consisting of representatives of the Ministry of Health (Population Directorate, Directorate of Epidemiology and Disease Control), the National School of Public Health and of Senior Consultants including the specialties Obstetrics, Neonatology and Endocrinology will be established and meet on a quarterly basis. There will be monthly encounters between principal investigators, local study coordinators and research assistants to supervise the health care facilities, discuss the progress of the study and monitor recruitment and follow up of participants. Adverse events will be closely followed-up and any unforeseen incidents reported to the advisory committee to take appropriate measures. Implementing partners at regional and district levels are the regional and both district medical officers. A local coordinator in each district will oversee the activities and assure monthly monitoring of the study sites.

### Data protection

The collected data will be kept under lock and key. Entered data will be stored in a password protected database. Throughout the trial, all patient and interview data will be handled confidential. Only the investigators will have access to the database.

## Discussion

Gestational diabetes screening and its initial management are fragmented in Morocco. Although general practitioners at health centers manage diabetic patients, the treatment of gestational diabetes is often reserved for specialists implying difficulties in access to higher levels of care and treatment delays. Implementation of a strategy that enables the primary health care sector to detect, initiate treatment and follow-up affected women has the potential to improve access to care, timely management and reduce obstetric and neonatal complications. The implication of the Moroccan Ministry of Health in the design and conduct of the study will be pivotal for the decision of a future scaling up. Although such a decision will be guided by acceptance, feasibility and clinical outcomes, the financial consequences cannot be omitted. Therefore an economic evaluation of the intervention is envisaged.

## Abbreviations

ANC: Antenatal Care
GA: Gestational Age
GDM: Gestational Diabetes Mellitus
OGTT: Oral Glucose Tolerance Test
